# Lives on the Line: The Online Lives of Girls and Women With and Without a Lifetime Eating Disorder Diagnosis

**DOI:** 10.3389/fpsyg.2018.02128

**Published:** 2018-11-01

**Authors:** Rachel Bachner-Melman, Einat Zontag-Oren, Ada H. Zohar, Helene Sher

**Affiliations:** ^1^Clinical Psychology Graduate Program, Ruppin Academic Center, Emek Hefer, Israel; ^2^Eating Disorder Unit, Child and Adolescent Psychiatry, Soroka University Medical Center, Beersheba, Israel

**Keywords:** women with ED, internet use, forums and blogs, eating disorders, online need satisfaction, online behaviors, online friends, social comparison

## Abstract

This study aimed to compare the scope, internet use patterns, and degree of online need satisfaction of girls and women with and without a lifetime eating disorder (ED) diagnosis. Participants were 122 females aged 12–30, 53 with a lifetime ED diagnosis recruited via a hospital-based treatment program, and 69 age-matched controls recruited via normative social media sites. Participants completed questionnaires assessing disordered eating, body image, positive and negative affect, general distress, and life satisfaction, and completed an online survey about the scope of their internet use, the frequency of watching and posting pictures and videos, online friendships and social comparison, fulfillment of needs online, and mood after internet use. All questionnaire scores differed significantly between groups in the expected directions. Whereas overall, ED and control groups spent similar amounts of time online (6.21, *SD* = 5.13), they spent this time differently. ED participants reported devoting 56.7% of their online time to eating, weight and body image, versus 29.1% for controls, and spent significantly more time than controls on forums and blogs (*t* = -5.3, *p* < 0.0001, Cohen’s *d* = 0.87). They also engaged more often in social comparison (*t* = 3.6, *p* < 0.005, Cohen’s *d* = 0.65), had a higher online–offline friend ratio (*t* = 3.7, *p* < 0.0001, Cohen’s *d* = 0.65), and more online friends with ED (*t* = 5.4, *p* < 0.0001, Cohen’s *d* = 0.89). In comparison to controls, ED participants reported that their use of forums and blogs gave them more eating- and weight-related advice, and a greater sense of belonging, social support, and safety resulting from anonymity, with effect sizes of 0.63–0.96. However, they also reported more negative affect after posting online. Most online behaviors and patterns correlated positively with measures of symptomatology and negatively with measures of psychological health, in both groups. Internet use was rarely addressed in therapy. Professionals, families and friends should help people with disordered eating and EDs to broaden the scope of their internet use. They should invest less in food- and weight-related forums/blogs, expand their “real life” social lives and develop their interpersonal skills, so that their legitimate needs can be satisfied face-to-face, rather than virtually. Clinicians should address the online lives of their ED clients in therapy.

## Introduction

Today’s young adults are “digital natives” (Prensky, 2001), who use the internet as a major information source, and conduct much of their social life online. In 2013–2014, 26.7% of global internet users were aged between 15 and 34 (Statista, 2017), and 18- to 29-year-old women were the heaviest social media users (Duggan and Brenner, 2013). Israeli youths engage online more than their American counterparts, with users aged 15–24 spending approximately one fifth of their waking hours online (Dror and Gershon, 2012). This study aimed to characterize the online lives of girls and young women with and without eating disorders (EDs).

Young people use the internet for many purposes. A major focus of shopping, fashion, celebrity, and magazine websites targeting adolescent girls and young women, is appearance, and internet exposure is associated with internalization of the thin ideal, body surveillance, and drive for thinness in adolescents (Tiggemann and Slater, 2013). Prospective research suggests that perceived pressure from the media to be thin predicts body dissatisfaction, which in turn predicts eating pathology (Stice, 2001). A recent meta-analysis suggests that the link between social media use and the internalization of the thin ideal in girls and women is explained by online appearance-related features (Mingoia et al., 2017). Social comparisons, so readily available online, are associated with body dissatisfaction, and people with EDs frequently compare their bodies to others’ (Fitzsimmons-Craft, 2017).

ED’s are negatively correlated with satisfying interpersonal relationships (Rastam et al., 2003). Since people with EDs often have trouble finding supportive social networks offline, online networking may have a particular hold for them. Different types of online social interaction are associated with different purposes and user experiences. Whereas social media users typically use their real names and photos, forum and blog users often comment and participate anonymously, removing or reducing social barriers to self-disclosure, minimizing perceived social threat and promoting a sense of belonging to a group of others with similar interests (Bargh and McKenna, 2004). On blogs, as in forums, fictive names are routinely used and posts can be read by non-registered people. Forums and blogs may therefore be particularly attractive to people with ED seeking to satisfy a range of needs online (Eichhorn, 2008; Ransom et al., 2010).

Social influence via the web can be powerful, both in destructive and in health-promoting ways. On the one hand, online therapeutic interventions (Traviss-Turner et al., 2017), recovery-orientated sites and forums (McCormack and Coulson, 2009), and prevention programs (Zabinski et al., 2003) can be empower people with ED to heal. On the other hand, pro-ED websites (Gale et al., 2016) and Twitter accounts (Bert et al., 2016) glorify and romanticize life-threatening malnutrition and provide guidelines for developing and maintaining symptoms. People with EDs, especially anorexia nervosa, are generally ambivalent toward recovery (Serpell et al., 1999), leading them to use both pro-recovery and pro-ED websites (Yom-Tov et al., 2012).

Online interaction serves people’s need for relatedness and social connection (Dunne et al., 2010). According to the “uses and gratifications” approach (Joinson, 2008), social interaction online provides opportunities for need gratification that may not be met offline. Whereas people seek to satisfy various needs online, the need for relatedness seems to be almost universal (Suler, 1999). In this study, three social needs (for social support, a sense of belonging, and safety), and an instrumental need, the need for personally relevant advice and information (here related to eating, weight and body image), were assessed:

(1) Social support: People with ED are often lonely (Levine, 2012) and need social approval (Bachner-Melman and Oakley, 2016). The web offers them an opportunity to feel less alone and receive social support at any time by communicating online with other people with EDs. Bonds between individuals with ED can be strong, and sometimes exclusive (Offord et al., 2006). Intense connections can create potential peer support either to overcome symptoms and move toward health (Federici and Kaplan, 2008), or to maintain and strengthen ED psychopathology (Gale et al., 2016).
(2) Sense of belonging: For people with psychological disorders, online forums provide a sense of community that helps them both avoid and cope with the stigmatization that unites them (Goffman, 1963). Social sensitivity, worry about appropriate social behavior, and fears of standing out and being judged are particularly common in people with ED (Bachner-Melman et al., 2009). Despite social disconnection, even during and after hospital stays, online friendships can provide an ongoing sense of stability and support (Patel et al., 2016), which often goes hand in hand with membership in groups of ill people who may have problematic or misguided cognitions and behaviors.
(3) Safety (privacy): When social interaction takes place online, particularly in forum or blog discussions, socio-demographic factors like age, race, and weight are minimized (Finfgeld, 2000). This enables people with ED, often socially isolated and/or stigmatized, to communicate and seek support online in relative anonymity, without revealing vulnerabilities directly to people in their lives. Shame, embarrassment, and fear are minimized and a sense of safety, privacy, and trust are maximized (Gale et al., 2016).
(4) Advice seeking is a central aim of internet use, achieved via both the social media (Dunne et al., 2010), and forums (Pendry and Salvatore, 2015). For people with ED, such information can include advice on therapy options, ‘tips’ on how to purge, and everything in between.

Awareness of and knowledge about the online activities of young people with ED has much to contribute to educators, parents, and health providers, who can help prevent emerging risk-related patterns or encourage change if they are entrenched. This study aimed to replicate and extend our knowledge about the online lives of girls and young women with a lifetime ED diagnosis (ED group; Eichhorn, 2008; Tong et al., 2013; Wolf et al., 2013; Mabe et al., 2014) and compare it to that of girls and young women never diagnosed with an ED (control group). It is, to our knowledge, the first quantitative study to compare the online behaviors of people with EDs to those of a control group, and the first empirical exploration of the online behaviors of women with EDs in Israel. Our hypotheses were as follows:

(1) The ED participants will score significantly higher on the EAT-26, BSQ, and PANAS negative affect, and significantly lower on the PANAS positive affect and SWLS than control women.
(2) ED participants will spend more time online than control participants and specifically, will spend more time on forums and blogs.
(3) ED participants will make greater use of the web, and of forums and blogs specifically, for ED-related issues than other control participants, and less use of the web for other purposes.
(4) ED participants will post and watch significantly more videos than controls online about eating, weight, and body image.
(5) ED participants will post less pictures of themselves and others online than controls, and will view more pictures posted by others.
(6) ED participants will have a higher online–offline friend ratio and more online friends with ED than control participants, and make more appearance-related comparisons online.
(7) ED participants will report that their needs for social support, belonging, safety, and information/advice are satisfied online, and specifically on forums and blogs, to a greater extent than control participants.
(8) ED participants will experience more negative emotion than control participants after being active online, in general and after using social media and forums/blogs for eating-, weight-, and body image-related issues.
(9) The online behaviors that differentiate ED from control participants will correlate positively with the severity of ED symptomatology, body dissatisfaction, depressive symptoms and negative affect, and negatively with positive affect and life satisfaction.

## Materials and Methods

### Participants

Participants in the study were 122 girls and women aged 12–30 (mean = 21.6 + 4.4). Fifty-three of the participants had a lifetime ED diagnosis (“ED group”; 41 anorexia nervosa, 1 bulimia nervosa, 3 binge ED, and 8 other specified EDs), and 69 (“control group”) reported never having an ED. ED participants were recruited via Soroka hospital, Beersheba, Israel and included inpatients and outpatients who had received a diagnosis of an ED on commencing treatment at the center and were in various stages of illness and recovery. Thirty-five (66%) of the ED participants were in treatment; 15 were inpatients and ten outpatients. The 18 (34%) not receiving treatment had been treated in the past; four as inpatients and fourteen as outpatients.

Control participants were recruited via Facebook groups of adolescents and young adults focusing on regional activities in the same area of Israel as the hospital. Participants without a good command of Hebrew were excluded. The population of Beersheba, the capital of an area in southern Israel called the Negev, is typical of the Israeli population. 96% (*n* = 117) of the participants were Jewish, of whom 70.5% (*n* = 86) defined themselves as secular, 14.8% (*n* = 18) traditional, and 14.8% (*n* = 18) religious. 10.7% (*n* = 13) were married. Three pairs of cinema tickets were raffled among participants and response rate in the ED group was approximately 70%. The groups did not differ significantly on any of the demographic variables assessed (see Table 1).

**Table 1:** Differences in demographic variables and questionnaire scores between women with and without a lifetime ED diagnosis.

	ED group	Control group	Significance	Effect size
	(*n* = 53) M (SD) or %	(*n* = 69) M (SD)	T/chi-square (*p*)	Cohen’s *d*
Age	21.92 (4.6)	21.31 (4.3)	NS	–
Marital status (single, in a relationship, married)	56.6%, 16.9%, 13.2%	58%, 33.3%, 8.7%	NS	–
Mother’s education (at least 1 university degree)	49.1%	68.1%	4.53 (*p* < 0.05)	–
Father’s education (at least 1 university degree)	45.3%	58%	NS	–
Level of religiosity (secular, traditional, religious)	59.6%, 19.2%. 21.2%	77.6%, 11.9%, 10.4%	NS	–
EAT-26	32.73 (17.04)	12.10 (14.47)	–6.56 (*p* < 0.0001)	–1.11
PANAS-SF-NA	3.23 (0.81)	2.31 (0.82)	–5.71 (*p* < 0.001)	–0.99
PANAS-SF-PA	2.97 (0.71)	3.31 (0.67)	2.53 (*p* < 0.05)	0.49
BSQ	4.60 (1.16)	2.67 (1.44)	–7.31 (*p* < 0.0001)	–1.18
SWLS	3.09 (1.47)	4.56 (1.43)	5.14 (*p* < 0.0001)	0.92

*EAT-26, Eating Attitude Test-26; PANAS-SF-NA, Negative Affect subscale of the Positive and Negative Affect Scale; PANAS-SF-PA, Positive Affect subscale of the Positive and Negative Affect Scale, Short Form; BSQ, Body Shape Questionnaire; SWLS, Satisfaction With Life Scale. *d* = 0.2 is considered a small effect size, *d* = 0.5 a medium effect size, and *d* = 0.8 a large effect size (Cohen, 1988, 1992).*

### Instruments

(1) Demographic and clinical questions: Participants were asked to report their age, marital status, parental level of education, and level of religious observance. In terms of clinical history, they reported height and weight, whether they had a current ED or had experienced an ED in the past. Those who had experienced an ED (past or present) reported diagnosis/es, age of onset, level of treatment (outpatient, day treatment, inpatient), number of hospitalizations and duration of treatment.
(2) Eating pathology was assessed using the Eating Attitudes Test-26 (EAT-26; Garner et al., 1983), a self-report questionnaire assessing disordered eating, e.g., “I feel extremely guilty after eating.” Responses are rated on a six-point Likert scale, with “never,” “rarely,” and “sometimes” scored 0, “often” 1, “usually” 2, and “always” 3. The EAT-26 is a widely used screening measures for EDs that captures variation in eating psychopathology in both clinical (Bachner-Melman et al., 2006) and non-clinical (Bachner-Melman et al., 2004; Rogoza et al., 2016) samples. A validated Hebrew translation was used (Koslowsky et al., 1992) and Cronbach’s alpha was 0.95.
(3) Positive and negative affect was measured by the Positive and Negative Affect Scale – Short Form (PANAS-SF; Thompson, 2007). This ten-item questionnaire includes five items assessing positive affect and five assessing negative affect. Participants rate the subjective strength of various emotions, e.g., excitement, anger during the past week, on a five-point Likert scale. A Hebrew translation previously employed in research (Zohar et al., 2011) was used. The validity and reliability of the PANAS-SF are acceptable in various cultures (Thompson, 2007) and in this study the alpha Cronbach for each of the subscales was 0.84.
(4) Satisfaction with life was measured using the Satisfaction With Life Scale (SWLS; Diener et al., 1985). The SWLS is comprised of five items requiring respondents to rate their general satisfaction on a seven-point Likert scale, e.g., “I am satisfied with my life.” The SWLS has good internal reliability (Diener et al., 1985), and the Hebrew translation used (Anaby et al., 2010) yielded an alpha Cronbach of 0.91.
(5) Psychological distress was assessed via the Brief Symptom Inventory-18 (BSI-18; Derogatis, 2001). The BSI-18 contains 18 items that include three subscales: Depression, Anxiety, and Somatization. Respondents are asked to indicate, on a five-point Likert scale, the severity of various symptoms, e.g., “feeling blue.” The BSI-18 has been shown to have good reliability (Derogatis, 2001), and the alpha Cronbach for the Hebrew translation used (Canetti et al., 1994) was 0.84 for Anxiety and for Somatization, and 0.86 for Depression. Helsinki approval for the BSI-18 was given for control participants only.
(6) Body image was assessed using a short, single-factor version of the Body Shape Questionnaire (BSQ-8C; Evans and Dolan, 1993). This 8-item questionnaire (e.g., “Have you felt excessively large and rounded?”) has good psychometric properties and high sensitivity (Pook et al., 2008). Responses are scored on a six-point Likert scale and the Hebrew translation used (Gigi et al., 2016) yielded an alpha Cronbach of 0.96.
(7) A 46-item survey about internet use devised for this study addressed.
  • General internet use (number of hours of use daily, and proportion of online time spent on different types of websites – five-point Likert scale from 1 [never] to 5 [almost all my online time]);
  • Internet use for eating, weight, and body image issues – percentage of total online time devoted to eating, weight and body image [0–100] and time allotted to eating, weight and body image on specific types of websites (five-point Likert scale from 1 [hardly any time] to 5 [almost all my time]);
  • Frequency of watching and posting videos and pictures (five-point Likert scale from 1 [almost never] to 5 [almost all my online time]);
  • Percentage of online friends with ED (five-point Likert scale from 1 [less than 20%] to 5 [over 80%]);
  • Ratio of offline to online friends (five-point scale from 1 [contact with friends mainly face to face and hardly via the internet] to 5 [contact with friends mainly via the internet and hardly face-to-face]);
  • Frequency of comparing online pictures of same-sex others with their own appearance (five-point Likert scale from 1 [almost never] to 5 [almost all the time]);
  • The extent to which social media sites and forums/blogs satisfy my need for 1. a sense of belonging (“feeling of belonging to a community you are part of”); 2. social support; 3. a sense of safety resulting from anonymity; 4. advice about eating, weight and body image (five-point Likert scale from 1 [hardly satisfies the need at all] to 5 [satisfies the need to a very great extent]);
  • Feelings experienced after being active online (in general), for example commenting, posting pictures and giving advice (sadness, relief, fear of others’ reactions, satisfaction as assessed by a five-point Likert scale from 1 [hardly any] to 5 [a lot]);
  • Mood after using social media sites and forums/blogs for eating-, weight-, and body-image issues (five-point Likert scale from 1 [very happy and encouraged] to 5 [very sad and frustrated]);
  • The degree to which internet use is/was discussed in therapy (ED group only, assessed on a five-point Likert scale from 1 [never] to 5 [all the time]).

The full survey is available from the corresponding author.

### Procedure

The study protocol and informed consent procedure were approved by the Ethics committee of Ruppin Academic Center and the Soroka Hospital Helsinki committee. Written informed consent was obtained from all adult participants and from the parents of all participants under the age of 18. Questionnaires were completed online by all participants and took approximately 40 min to complete. Control participants and ED participants who were not hospitalized completed them at their own convenience. The 15 participants in inpatient treatment completed the questionnaires online under the supervision of a research assistant. Since the inpatient participants had limited access to online material while in the ward, they were asked to report on their internet use during the period preceding their hospitalization. The data were entered electronically into Qualtrics (2002) and analyzed using SPSS version 21.

Statistical analyses: Groups were tested for homogeneity by comparing demographic and clinical variables. Analysis of variance was used to compare the ED group with the controls on the study variables, after determining whether or not variance was equal in both group and proceeding accordingly. Whenever differences were statistically significant, Cohen’s d was calculated to estimate effect size. Pearson correlations were calculated between the study variables.

## Results

### Group Comparisons

To test hypothesis no. 1, scores on the EAT-26, BSQ, I-PANAS-SF and SWLS were compared between groups using *t*-tests. Since the ED sample was composed of 41 participants with a lifetime diagnosis of AN, and 12 with a lifetime diagnosis of other EDs, we compared these two subgroups using *t*-tests for all variables. Since no significant differences were observed, we combined these subgroups into one ED group. As expected, these ED women scored significantly higher on the EAT-26, BSQ, and PANAS negative affect, and significantly lower on the PANAS positive affect and SWLS than control women (see Table 1). Thirty-two (72%) of the ED participants and 11 (18%) of the control participants scored above the clinical cutoff (19) (Garner et al., 1984). We decided not to exclude control participants who scored above the EAT-26 cutoff, since this cutoff has been found to incorrectly classify 16.4% of respondents (Garner et al., 1984), and because excluding individuals with a tendency toward significant psychopathology results in a “super-normal” control group (Kendler, 1990).

### General Internet Use

Participants spent a mean of 6.21 (*SD* = 5.13) hours online every day, with no significant difference between the ED group (mean = 5.64, *SD* = 4.21) and the control group (mean = 6.92, *SD* = 6.1). However, ED participants reported spending more of their online time (mean = X hours, SD = Y) than control participants on forums and blogs (*t* = -5.3, *p* < 0.0001, Cohen’s *d* = 0.87) and significantly less seeking knowledge (*t* = 2.06, *p* < 0.05, Cohen’s *d* = 0.38), keeping up with the news (*t* = 2.34, *p* < 0.05, Cohen’s *d* = 0.42), and shopping (*t* = 2.29, *p* < 0.05, Cohen’s *d* = 0.41). Hypothesis no. 2 was therefore partially confirmed.

### Internet Use for Eating, Weight, and Body Image Issues

Eating disorder participants reported devoting a mean of 56.7% of their total online time to eating, weight, and body image, significantly more than control participants, who reported devoting 29.1% to these issues (*t* = 5.1, *p* < 0.0001, Cohen’s *d* = 0.87). Specifically, ED participants reported that they devoted more of their time using forums and blogs to eating, weight and body image issues than controls (*t* = 5.1, *p* < 0.0001, Cohen’s *d* = 0.85), but this was not true for social media, music and video, educational, shopping or news sites. Hypothesis no. 3 was therefore confirmed.

### Watching and Posting Videos

Eating disorder participants watched significantly more videos than controls online about eating, weight, and body image (*t* = 5.6, *p* < 0.001, Cohen’s *d* = 0.96) and less humorous videos (*t* = 3.0, *p* < 0.005, Cohen’s *d* = 0.57). However, there was no group difference in the frequency of posting or uploading videos. Hypothesis no. 4 was therefore partially confirmed.

### Watching and Posting Pictures

Eating disorder participants were less likely to post pictures of themselves and others online than controls (*t* = 3.1, *p* < 0.005, Cohen’s *d* = 0.58). However, both groups were equally likely to view pictures posted by others. Hypothesis no. 5 was therefore partially confirmed.

### Friendships and Social Comparison

Eating disorder participants reported a greater ratio than controls of online to real-life friends (*t* = 3.7, *p* < 0.0001, Cohen’s *d* = 0.65), and a larger percentage of online friends with ED (*t* = 5.4, *p* < 0.0001, Cohen’s *d* = 0.89). ED participants reported comparing online pictures of same-sex others with their own appearance significantly more often than controls (*t* = 3.6, *p* < 0.005, Cohen’s *d* = 0.65). Hypothesis no. 6 was therefore confirmed.

### Reported Fulfillment of Needs Online

Participants were asked to indicate on a five-point Likert scale to what extent their use of (a) social media sites and (b) forums/blogs provided them with (i) a sense of belonging; (ii) social support; (iii) a sense of safety resulting from anonymity, and (iv) advice about eating, weight and body image. As can be seen from Table 2, girls and women with a lifetime ED diagnosis reported that on social media sites they felt more safety due to anonymity than controls, and received more advice about eating and weight issues, but did not experience more social support or a greater sense of belonging. However, the ED group reported that on forums and blogs, all four needs were significantly more satisfied than for controls, with effect sizes of 0.63–0.96. The effect sizes for the difference in advice received about eating, weight and body image were very large both for the social media and for forums/blogs (Cohen’s *d* = 0.84 and 0.96, respectively). Hypothesis no. 7 was therefore largely confirmed.

**Table 2:** Differences between ED group (*n* = 53) and control group (*n* = 69) in the degree to which internet use was reported to fulfill needs.

Need (online source)	Group	Mean (SD)	Significance T (*p*)	Effect size Cohen’s *d*
Sense of belonging (social media)	ED	2.96 (1.19)	NS	0.07
	Control	2.88 (1.19)		
Sense of belonging (forums and blogs)	ED	2.90 (1.34)	4.44 (*p* < 0.0001)	0.78
	Control	1.88 (1.11)		
Social support (social media)	ED	2.96 (1.14)	NS	0.15
	Control	2.78 (1.19)		
Social support (forums and blogs)	ED	3.14 (1.32)	4.50 (*p* < 0.0001)	0.79
	Control	2.08 (1.19)		
Sense of safety (social media)	ED	2.75 (1.33)	2.46 (*p* < 0.5)	0.45
	Control	2.18 (1.18)		
Sense of safety (forums and blogs)	ED	2.92 (1.40)	3.49 (*p* < 0.001)	0.63
	Control	2.05 (1.25)		
Advice on eating and weight (social media)	ED	3.45 (1.40)	4.98 (*p* < 0.0001)	0.84
	Control	2.25 (1.24)		
Advice on eating and weight (forums and blogs)	ED	3.35 (1.32)	5.77 (*p* < 0.0001)	0.96
	Control	1.98 (1.19)		

*d = 0.2 is considered a small effect size, *d* = 0.5 a medium effect size, and *d* = 0.8 a large effect size (Cohen, 1988, 1992).*

### Mood After Internet Use

After being active online (in general), for example commenting, posting pictures, and giving advice, ED participants reported feeling sadder than control participants (*t* = 2.73, *p* < 0.01, Cohen’s *d* = 0.52), but there was no difference between the groups in the degree to which they experienced relief, fear of others’ reactions and satisfaction from having contributed something positive.

Eating disorder participants reported feeling significantly sadder and more frustrated than controls (*t* = 2.71, *p* < 0.01, Cohen’s *d* = 0.49) after using social media for eating-, weight-, and body image-related issues, and even more significantly sadder and more frustrated than control participants after using forums/blogs for these purposes (*t* = 4.34, *p* < 0.0001, Cohen’s *d* = 0.77). Hypothesis no. 8 was therefore largely confirmed.

### Correlations Between Survey Items and Measures of Symptomatology and Psychological Health

Table 3 shows the pattern of correlations between survey items and questionnaire scores. Both the direction and the power of the correlations were very similar in both groups and tended to be slightly but not significantly stronger in the control group. Correlations are therefore presented for the entire sample, with the exception of the BSI-18 (control group only) and items not completed by controls (ED group only). Strikingly, most of the survey items characteristic of ED participants correlated significantly and positively with EAT-26, BSQ, and PANAS-NA scores and negatively with PANAS-PA and SWLS scores in the combined sample. Correlations with PANAS-PA scores were the weakest and least consistent. Most items also correlated negatively with BSI-18 scores within the control group. Items relating to the use of forums/blogs, but not the use of social media sites, tended to show this correlational pattern. Watching ED-related videos, using the internet to strengthen ED symptoms, having predominantly online friends, comparing one’s appearance to others’, need fulfillment on forums/blogs, and negative mood after posting and commenting online were also all robustly correlated with the measures of symptomatology and psychological health administered in the expected directions. Hypothesis no. 9 therefore confirmed.

**Table 3:** Pearson correlations between survey items and measures of symptomatology and psychological health (*N* = 122).

Survey item	EAT-26	BSQ	NA	PA	SWLS	^†^BSI-18
% of online time devoted to ED content	0.68**	0.63**	0.42**	–0.10	–35^∗∗^	0.54**
Frequency of use of forums/blogs	0.51**	0.50**	0.44**	–0.21*	–42^∗∗^	0.40**
Frequency of use of social media sites	0.20*	0.19	0.18	–0.05	–0.17	0.20
Link between forum/blog use and eating/weight/body image	0.46**	0.42**	0.42**	–0.13	–0.22*	0.43**
Link between social media use and eating/weight/body image	0.17	0.16	0.10	–0.05	0.02	0.11
Frequency of posting pictures of self/others	–0.10	–0.11	–0.20*	0.26**	0.22*	–0.12
Frequency of watching ED-related videos	0.56**	0.52**	0.37**	–0.24*	–29^∗∗^	0.24
Frequency of watching funny videos	–0.32**	–0.23*	–0.23*	0.16	0.19	–0.14
Ratio of online to offline friends	0.43**	0.40**	0.44**	–32^∗∗^	–33^∗∗^	0.46**
Comparing online pictures of other girls/women with own appearance	0.53**	0.62**	0.47**	–0.23*	–32^∗∗^	0.52**
Use of internet to strengthen symptoms^‡^	0.50**	0.43**	0.35*	–0.11	–0.30*	–
Use of internet to recover^‡^	–0.08	–0.06	–0.07	0.06	0.08	–
Perceived social support on forums/blogs	0.48**	0.40**	0.37**	–0.15	–30^∗∗^	0.33*
Sense of belonging on forums/blogs	0.52**	0.41**	0.40**	–0.12	–34^∗∗^	0.27*
Sense of safety on forums/blogs	0.47**	0.31**	0.27**	–0.24*	–30^∗∗^	0.19
Advise/tips received on forums/blogs	0.64**	0.58**	0.49**	–25^∗∗^	–43^∗∗^	0.44**
Advise/tips received on social media	0.46**	0.45**	0.36**	–0.16	–33^∗∗^	0.34**
Negative mood after posting/commenting	0.52**	0.58**	0.45**	–26^∗∗^	–33^∗∗^	0.44**

*^∗^*p* < 0.05; ^∗∗^*p* < 0.01 two tailed significance test. ^†^Data available for control participants only (*n* = 69), because the ED participants did not complete the BSI-18. ^‡^Data available for ED participants only (*n* = 53). EAT-26, Eating Attitudes Test-26; BSQ, Body Shape Questionnaire; NA, Negative Affect subscale of the PANAS; PA, Positive Affect subscale of the PANAS; SWLS, Satisfaction With Life Scale; BSI-18, Brief Symptom Inventory-18.*

### Discussion of Internet Use in Therapy

Eating disorder participants were asked to rate how often their internet use had been raised in therapy between 1 (never) and 5 (all the time). The average score was 1.82 (*SD* = 1.02), falling between “never” and “occasionally.” Thirty-four (77%) of respondents replied “never” or “occasionally,” seven (16%) “sometimes,” and only three (7%) “often” or “all the time.” The correlation between the frequency with which internet use was spoken about in therapy and the percentage of online time devoted to eating, weight, and body image was 0.36 (*p* < 0.05).

## Discussion

This study adds to the growing body of research on the internet use of people with EDs and expands it by comparing the web use of girls and women with and without an ED history. Both ED and control participants reported spending over one third of their waking hours online. The lack of difference between time spent online by ED and control participants corroborates previous findings that online hours are not associated with psychopathology (Harris et al., 2017). However, ED participants reported devoting significantly more of their online time than controls to ED-related content. They spent more time on forums/blogs, and less time on information, news and shopping sites. They reported focusing more on ED-related issues than controls when using forums and blogs, but not when using social media, music and video, educational, shopping or news sites. Overall, the online world of ED participants was impoverished in comparison to healthy controls, focusing narrowly on the world of EDs to the detriment of general knowledge, cultural issues, humor, and broader aspects of life and living.

The differences that emerged between traditional discussion forums and the more mainstream social media provides evidence for their different functions that have implications for their use by women with ED symptoms. Our hypothesis that a sense of safety and privacy on forums and blogs would be more salient for ED than for control participants was supported. The need for privacy on the web is a universal concern (Nissenbaum, 2011) and online anonymity reduces a sense of threat and fosters a feeling of belonging to a virtual group (Bargh and McKenna, 2004). The virtual connections girls and women with ED symptoms seem to form specifically on forums/blogs seem to be meaningful and to contribute to a sense of belonging and of social support.

The need for social support and a sense of belonging is healthy and normative, especially during adolescence (Blakemore and Mills, 2014), and identification with a social group is strongly linked to health and well-being (Haslam and Reicher, 2006). Yet the fulfillment of the social needs examined in this study was clearly associated with negative outcomes for people with EDs and disordered eating, especially in the context of forums and blogs. Belonging to a group can have beneficial effects if the group positively influences its members. However, communities of people with EDs over-value thinness and share a desire to lose weight (Harper et al., 2008). Pro-ED groups openly provide guidance and encouragement for a destructive way of life, mindset and behaviors (Borzekowski et al., 2010). The price tag of a yearned sense of support and belonging therefore seems to be the adherence to destructive and pathological norms.

Eating disorder participants also sought and received ED-related information and advice more often than controls, both on forums/blogs and the social media. As with support and belonging, receiving information and advice can be beneficial if the content of this advice is constructive. However, seeking advice about weight, eating and body image online is a dangerous pursuit, because the boundaries between pro-ana websites and ‘routine,’ societal endorsement of thinness are becoming more and more blurred (Cobb, 2017). Receiving advice about unhealthy but “effective” weight loss strategies, for example, clearly undermines health and well-being. Indeed, for both groups, receiving ED-related advice online was positively associated with measures of symptomatology and negatively with psychological health. Together, the current findings present a picture compatible with research showing online need satisfaction to be associated positively with pathological internet use and negatively with need satisfaction offline (Deng et al., 2012; Shen et al., 2013).

Eating disorder participants compared themselves more often than controls to online pictures of others, supporting research on social comparison (Bauer et al., 2017). The links we observed between appearance-focused comparisons, eating symptomatology, affect and life satisfaction have been previously observed (Fitzsimmons-Craft et al., 2016), supporting a continuum approach to EDs. ED participants tended to post pictures of themselves and others less frequently than controls, possibly reflecting a lack of comfort sharing identifiable, appearance-focused content with others. ED participants also reported having more online friends with ED than controls, which can play a role in maintaining the illness (Patel et al., 2016), and having a higher ratio of online to offline friends.

DiMaggio et al. (2001) suggested that online interactions can be positive if they supplement, rather than replace, face-to-face social interaction. Jetten et al. (2009) warned that “virtual-world networking can become a substitute for real-world engagement” (p. 33), and Turkle (2012) argued that virtual relationships are superficial, require little emotional investment, and prevent people from nurturing ‘real’ connections offline. These claims seem pertinent to participants in this study, whose reported ratio of online to offline friends correlated in both groups with measures of symptomatology and psychological health. Moreover, ED participants reported more negative mood than controls after using the internet, and mood level was associated with measures of symptomatology and psychological health in both groups. The online fulfillment of social needs therefore seems, overall, to negatively impact the well-being of participants with ED symptomatology, despite their high levels of reported online need fulfillment. Although online communication may help people with ED to remain in contact with others and overcome isolation to some extent (Patel et al., 2016), our findings support the view that online friendships constitute a defensive strategy that strengthens avoidance of face-to-face contact (Nie and Hillygus, 2002; Doris et al., 2014). Even though EDs are associated with a lack of “real-life” social connectedness, recovery is associated with improved relationships (Zohar et al., 2016).

The survey administered in this study asked about “using the internet to strengthen ED symptoms,” an indirect way of asking about using pro-ED sites. Our results replicate previous findings that pro-ED website usage is associated with the severity of ED symptoms (Peebles et al., 2012). They also extend them by showing that use of the web by girls and women with a lifetime ED diagnosis for purposes of strengthening ED symptoms is positively associated with disordered eating, body dissatisfaction, and negative affect, and negatively associated with life satisfaction. Interestingly, use of the web for recovery purposes was unrelated to these variables. This may be because intention to recover reflects a snapshot in time devoid of the process ahead, and/or because declarations of motivation to recover from an ED seem unrelated to actual behavioral recovery (Waller, 2012).

Our results show that girls and women with a current or past ED tend to use the internet to focus on eating, weight, and body image, to use forums and blogs, to use the internet to satisfy their needs, to have a high ratio of online to offline friends, to compare their appearance with others’ online pictures, and to experience negative mood after using the web. This pattern of web use was positively associated with the severity of ED symptoms, body dissatisfaction, and negative affect and negatively associated with satisfaction with life and, to a lesser extent, positive affect. Importantly, these associations were found not only for the ED group, but for a group of women who had never been diagnosed with an ED as well, providing support for the validity of an ED continuum (Tylka and Subich, 1999). In this non-clinical group, these behaviors also correlated with psychological distress. Therefore, this coupling of detrimental internet use with poor psychological health is valid for all young women, regardless of whether or not they have experienced an ED. All would gain, it seems, from leading online lives that do not focus on eating, weight, and body image.

This study has several limitations. First, the sample was comprised of a small number of young, Jewish, Israeli females, and most ED participants had anorexia nervosa. Results may not be generalizable to males, other age groups, people with other ED diagnoses, and people from different cultures. Second, data on comorbid disorders was not available. Third, online behaviors are not always reported accurately (Sharkow, 2016), so results should be viewed with caution. Fourth, findings require replication, since we are unaware of other studies comparing the functions of forums/blogs and the social media for people with ED. Fifth, blogs and forums were examined together, but may also fulfill separate ED-related functions. Finally, results do not indicate whether the online patterns observed lead to ED pathology, ED pathology leads to internet use, or both.

Our results have practical and therapeutic implications. Since internet use focusing on eating, weight, and body image was associated with similar degrees of distress and dysfunction for both clinical and non-clinical groups, online behaviors and patterns could be targeted in prevention programs. ED clinicians should actively ask about what their clients are doing online and address this in therapy. Parents too, should be informed about and take an interest in the eating- and body image-related internet options available to young people today and be on the alert for possible signs of their negative influence. Therapists should also help their clients with ED, who tend to have impaired social functioning (Tchanturia et al., 2012), to develop their “real life” social skills. Interventions such as social problem-solving, interpersonal effectiveness skills, and facial expression recognition (Davies et al., 2016) for example, could help them connect better with healthy peers offline. Parents, therapists and others should guide youngsters with EDs, disordered eating or body image problems to maintain a health-enhancing online social presence that supplements, but does not replace, face-to-face interactions and does not encourage them to avoid conflicts that need to be met. Additionally, decreasing stigma toward people with EDs (Murakami et al., 2016) may encourage them to create connections with healthy people around them and speak about their disorders, reducing their need to do this mainly or exclusively online.

Future research should investigate the online behaviors of males with ED. It should also examine whether a propensity to spend online time on pathology-focused forums and blogs is ED-specific. For example, perhaps other shy, socially withdrawn populations (Orr et al., 2009) such as people with social anxiety disorder or obsessive-compulsive disorder use the internet in similar ways to people with ED. In addition, changes that could tap into the positive potential of forums/blogs should be investigated, for example forum moderation (Kendal et al., 2017). Finally, since this study was cross-sectional, no conclusions can be made about how the online lives of people with EDs change with recovery. Whereas results support the assumption that as symptoms decrease and life satisfaction increases during the recovery process, this question should be addressed empirically.

## Author Contributions

RB-M designed the study together with EZ-O, oversaw all aspects of the study, and wrote most of the manuscript. EZ-O (equal first author) conceived, designed and conducted the study, and contributed to the data analysis and writing of the manuscript. AZ contributed significantly to the data analysis and the writing of the manuscript, especially the results. HS oversaw the study, diagnosed and recruited most of the participants with a lifetime ED diagnosis, who were past and present patients in the ward that she heads. She also contributed to the writing of the manuscript.

## Conflict of Interest Statement

The authors declare that the research was conducted in the absence of any commercial or financial relationships that could be construed as a potential conflict of interest.
